# Spatial variation in leopard (*Panthera pardus*) site use across a gradient of anthropogenic pressure in Tanzania's Ruaha landscape

**DOI:** 10.1371/journal.pone.0204370

**Published:** 2018-10-10

**Authors:** Leandro Abade, Jeremy Cusack, Remington J. Moll, Paolo Strampelli, Amy J. Dickman, David W. Macdonald, Robert A. Montgomery

**Affiliations:** 1 Wildlife Conservation Research Unit, Department of Zoology, The Recanati-Kaplan Centre, Tubney, Abingdon, United Kingdom; 2 Research on the Ecology of Carnivores and Their Prey Laboratory, Department of Fisheries and Wildlife, Michigan State University, East Lansing, Michigan, United States of America; 3 University of Stirling, Stirling, United Kingdom; Université de Sherbrooke, CANADA

## Abstract

Understanding large carnivore occurrence patterns in anthropogenic landscapes adjacent to protected areas is central to developing actions for species conservation in an increasingly human-dominated world. Among large carnivores, leopards (*Panthera pardus*) are the most widely distributed felid. Leopards occupying anthropogenic landscapes frequently come into conflict with humans, which often results in leopard mortality. Leopards’ use of anthropogenic landscapes, and their frequent involvement with conflict, make them an insightful species for understanding the determinants of carnivore occurrence across human-dominated habitats. We evaluated the spatial variation in leopard site use across a multiple-use landscape in Tanzania’s Ruaha landscape. Our study region encompassed *i*) Ruaha National Park, where human activities were restricted and sport hunting was prohibited; *ii*) the Pawaga-Idodi Wildlife Management Area, where wildlife sport hunting, wildlife poaching, and illegal pastoralism all occurred at relatively low levels; and *iii*) surrounding village lands where carnivores and other wildlife were frequently exposed to human-carnivore conflict related-killings and agricultural habitat conversion and development. We investigated leopard occurrence across the study region via an extensive camera trapping network. We estimated site use as a function of environmental (i.e. habitat and anthropogenic) variables using occupancy models within a Bayesian framework. We observed a steady decline in leopard site use with downgrading protected area status from the national park to the Wildlife Management Area and village lands. Our findings suggest that human-related activities such as increased livestock presence and proximity to human households exerted stronger influence than prey availability on leopard site use, and were the major limiting factors of leopard distribution across the gradient of human pressure, especially in the village lands outside Ruaha National Park. Overall, our study provides valuable information about the determinants of spatial distribution of leopards in human-dominated landscapes that can help inform conservation strategies in the borderlands adjacent to protected areas.

## 1. Introduction

As apex predators, large-bodied mammals of the order *Carnivora* can exert important influence on regulation of trophic interactions and the maintenance of ecosystem functions [1, 2]. Large carnivores, besides their intrinsic value as species [3], are also important revenue-generators for a multimillion-dollar ecotourism and sport hunting industry that contributes to national economies as well as the conservation and management of wildlife and wilderness, particularly in Africa [4, 5]. Despite clear ecological, economic, and intrinsic value, large carnivore populations are threatened globally, with 24 of the remaining 31 species documented to be declining [1]. Such population losses are attributable to habitat conversion, human persecution, prey depletion, unsustainable hunting, and exploitation for body parts [1, 6, 7]. Human population growth and urbanization around protected areas, especially in the sub-Saharan African countries [8, 9], present imminent challenges for carnivore conservation. For example, mortality is higher along the boundaries of protected areas where large carnivores risk being killed preventatively or in retaliation to predation events that can cause substantial financial loss to people’s livelihoods [10–12]. The habitats associated with this human-carnivore interface can function as population sinks, whereby the high human-induced large carnivore offtake can “drain” populations from the bordering protected areas and compromise population persistence [11, 12]. However, as large carnivores are often wide-ranging and maintain large home ranges [1, 13], they usually rely on these peripheral human-dominated lands around protected areas [11, 14, 15] that can provide important habitats for these species. For instance, 68% of the most suitable habitats for leopards in South Africa have been estimated to occur outside national parks and protected areas, in areas of human occupation and subject to habitat conversion [16]. Thus, these human-dominated habitats can be essential to the conservation of large carnivore populations [10, 11, 17, 18]. Accordingly, determining the extent to which large carnivores can occupy areas of increasing human pressure, such as those represented by human encroachment of wildlands and agro-pastoralism, is of major importance for their conservation.

Among large carnivores, leopards (*Panthera pardus*) are the most widespread felid species, occupying the most diverse habitat types including deserts, forests, and savannahs [19]. Leopards’ behavioural flexibility and dietary plasticity facilitates their successful occupation of highly modified and heavily disturbed human-dominated landscapes, given adequate human tolerance to their presence in such habitats [20, 21]. For instance, even in densely populated areas (400 people/km^2^) leopards can live alongside people by mostly feeding on livestock and domestic dogs, and finding refuge in crops and agricultural lands [20, 21]. Despite such ecological plasticity, leopards are threatened by rampant habitat destruction and fragmentation, prey depletion induced by bushmeat poaching and overgrazing, unsustainable harvest by sport hunting and to attend demands for body parts, and conflict-related mortality [19, 22, 23]. As a result, leopard populations have experienced >30% global range contraction in the past 20 years. In Africa, leopards have lost 48–67% of their historical distribution, with the most pronounced reductions in northern and western Africa [19]. The species is expected to undergo further population decline across its overall Sub-Saharan African range given the observed high rate of prey depletion [22] and habitat loss induced by increasing human population in the next 50 years [23].

Tanzania is one of the most important countries for leopard conservation in Africa, where its vast array of national parks and game reserves protects substantial portions of the leopard’s extant range [19]. Leopards represent an important economic asset for Tanzania, as the species is among the most exported trophy species; in 2008 hunting of leopards and other mammalian megafauna contributed to a USD 56.3 million revenue for hunting operators and governments [4]. Despite the ecological and economic importance of leopards, the current lack of empirical field data on leopard ecology hinders the development of effective conservation strategies designed to protect the species in Tanzania [24, 25].

In this study, we investigated the factors affecting the probability of leopard site use at the interface of protected and unprotected habitat in southern Tanzania’s Ruaha landscape. Our study area encompassed the eastern portions of Ruaha National Park, the adjacent semi-protected Pawaga-Idodi Wildlife Management Area (WMA), and unprotected village lands. Specifically, we assessed spatial variation in leopard site use in response to (*i*) anthropogenic disturbance, as indicated by distance to households and livestock number, (*ii*) the availability of primary prey species, and (*iii*) proximity to water sources. Documenting the factors associated with carnivore site use is central to prioritising conservation efforts for these species. The methods and framework presented in this study provide a timely and useful tool that is going to become ever more important in increasingly human-modified protected to unprotected habitat interfaces.

## 2. Material and methods

### Ethics statement

Data collection was based on the use of camera traps, a non-invasive method that does not involve contact with the study species, nor interfere with their natural behaviour. Fieldwork was carried out under research permit no. TWRI/TST/65/VOL.VII/85/146 to LA, issued by the Tanzania Wildlife Research Institute (TAWIRI) and the Commission for Research and Technology (COSTECH).

### The Ruaha landscape

**Fig 1:**
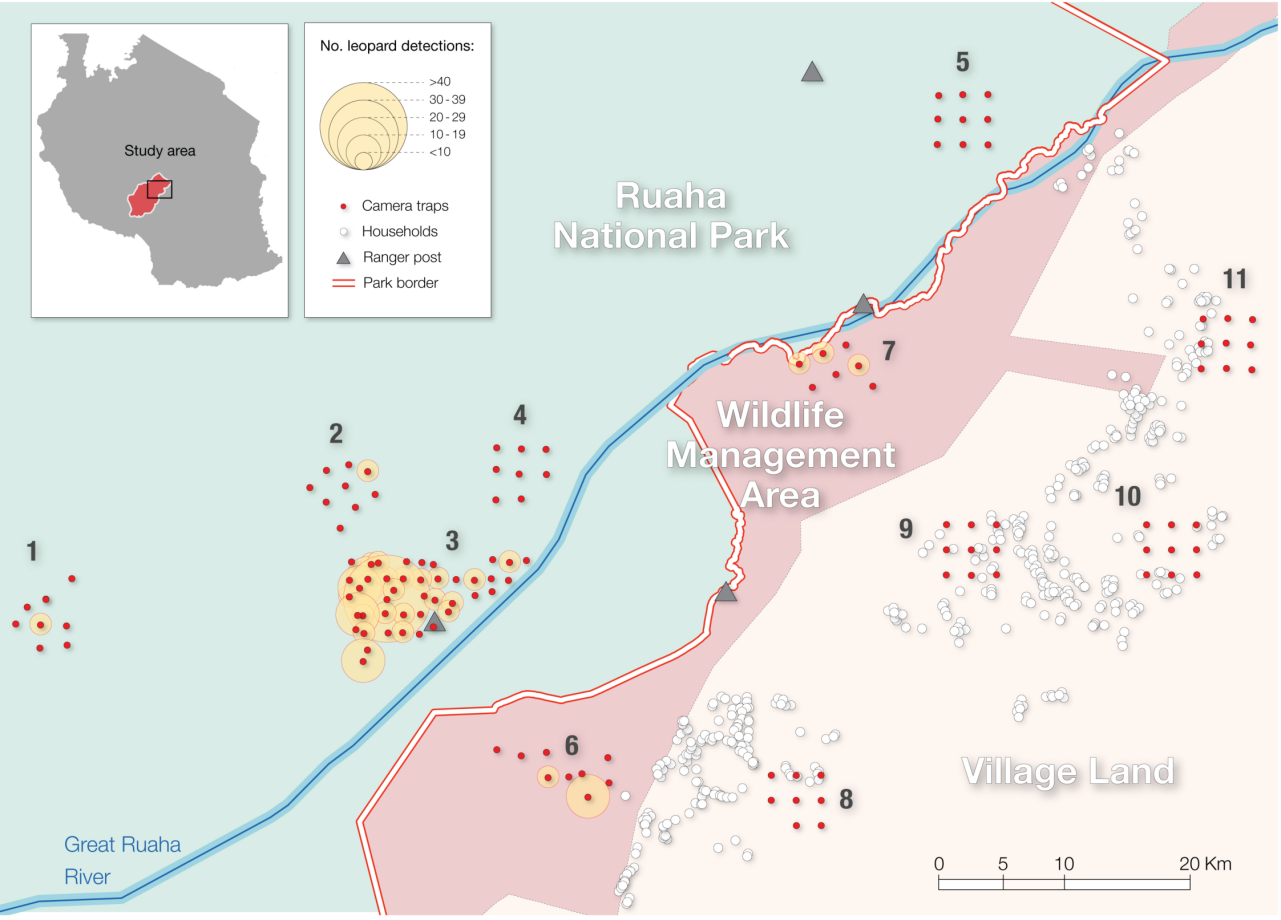
Spatial distribution of the camera-trap stations (red shaded circles) across the Ruaha landscape. 1–11 represents sampling areas: 1. Mdonya; 2. Kwihala; 3. Msembe; 4. Mwagusi; 5. Lunda-Ilolo; 6. Pawaga; 7. Lunda; 8. Idodi; 9. Malinzanga; 10. Nyamahana; 11. Magosi. The yellow shaded circles represent the number of independent detections of leopards (*Panthera pardus*) at each camera-trap station (> 5 minutes between detection).

We conducted our study in southern Tanzania across the Ruaha landscape (Fig 1). The Ruaha landscape spans over 50 000 km^2^ and supports substantial populations of large carnivores. For this reason, the landscape has been listed by the Tanzania Wildlife Research Institute as a priority for carnivore research and conservation [25]. Ruaha National Park is one of the largest national parks in Africa, spanning over 20 226 km^2^. Trophy hunting of wildlife is prohibited within the park and in the village lands, but is permitted in limited sections of the WMA. In the village lands, large carnivores are exposed to anthropogenic disturbance, including intense human-carnivore conflict, bushmeat snaring, and indiscriminate poisoning. The village lands are inhabited by over 60 000 people divided among 21 villages [26]. The predominant livelihood is agropastoralism [27]. Donkeys, goats, and cattle are the most commonly kept domestic livestock. Although no official numbers for livestock abundance are available for this area, the overall Iringa region, within which Ruaha National Park sits, contains a fifth of Tanzania’s total domestic animals, with > 620 000 livestock and > 1.5 million poultry [27]. Attitudes towards large carnivores among village members tend to be negative, motivated by both real and perceived carnivore depredation of livestock [28]. Consequently, large carnivores experience high rates of human-induced mortality in this landscape. From 2010–2016, 100 lions and other large carnivores were killed by people. Given the intense conflict and mortality rates, and the paucity of information about the spatial distribution of large carnivores in these areas [24, 25], it is imperative to improve understanding of the ecological and anthropogenic factors influencing large carnivore occurrence in these areas.

The climate of the region is semi-arid to arid, with an average annual precipitation of 500 mm, and a bimodal rainy season from December to January and March to April [29]. The vegetation cover is a mosaic of semi-arid savannahs and northerly Zambesian *miombo* woodlands [30]. The village lands are primarily covered by agricultural fields (mostly rice and maize crops), and livestock grazing areas. The Greater Ruaha River is the main water source in the study area, especially during the dry season. This river provides key resources for wildlife, attracting species towards the park borders with the WMA and village land.

### Leopard data

To document leopard site use, we deployed 127 non-baited, remotely triggered, single camera-trap stations (CTs) that sampled 11 areas across the Ruaha landscape during the dry seasons of 2014 and 2015. In 2014, we placed 42 Reconyx HC500 CTs along animal trails, and sampled the Msembe area, near the park headquarters, where there is low anthropogenic pressure [31]. In 2015, we used 85 Bushnell Scoutguard CTs and extended sampling to other 10 areas, including four sampling areas in RNP, two in the WMA, and four in the village lands (Fig 1). We used a pseudostratified method for deploying our CTs, ensuring a minimum 1.5–2 km distance between stations, and 15–20 km distance between sampling areas whenever possible. The sampling areas were distributed across a range of distances from the border of the national park (0–10 km; 10–20 km; >30 km) to enable examining potential spatial variation in leopard occurrence (Fig 1). We set the CTs facing animal trails when the pre-defined GPS coordinates were found within 5 meters from the nearest open path showing signs of animal use. We adopted this design so as to increase detection of more elusive species [32]. All the CTs were placed in trees or poles at a height of 0.3–0.5 meters off the ground. We visited the CTs every 30–50 days to retrieve data and service the traps. Though certain regions of the national park were inaccessible (especially in the road-less southern sections), our CTs placement intended to capture substantial habitat heterogeneity observed across the landscape.

We pooled the overall data and analysed it in a single-season framework, as previous studies conducted in the central areas of the Ruaha National Park have found large carnivores to have similar site use patterns across the dry seasons of 2014 and 2015 [31]. We collapsed the temporal extent of the sampling into seven days bin intervals, across a 32-week survey (~210 days) period. This timeframe has been chosen to ensure continued sampling through the whole dry season. Given the long duration of our survey across the whole dry seasons, we were unable to meet the population closure assumption of the occupancy model [33–35]. However, such assumption can be relaxed when changes in the population of interested are assumed to happen randomly during the survey period [34], which may the case with our extended sampling period. The relaxation of the population closure assumption requires changing the interpretation of the occupancy parameter from true occupancy to proportion of site used by the species, which originally was our main interest. Thus, in this study, site use equates to the probability that a given site was used during the overall survey period, rather than the probability of continuous site occupation [35].

The leopard occurrence data used for the model can be found freely available at https://github.com/labade/GitHub/tree/master/leop_occu_data.

### Environmental covariates

We modelled leopard site use as a function of five environmental covariates known to influence leopard habitat selection (Table 1) [36–40]. We calculated the distance to Great Ruaha River and distance to household covariates as rasters at a resolution of 500 m (S1 Fig). We generated the rasters in QGIS 2.6.0 [41] from freely available geoprocessed satellite imagery and data collected by University of Oxford’s Wildlife Conservation Research Unit, Ruaha Carnivore Project. We developed a primary wild prey availability covariate for leopards. To do so, we calculated a temporal catch-per unit effort (CPUE) index of prey availability for each CTs based on the number of independent records for the main five leopard prey species [42]. Prey species included bushbuck (*Tragelaphus scriptus*), common duiker (*Sylvicapra grimmia*), greater kudu (*Tragelaphus strepsiceros*), impala (*Aepyceros melampus*), and warthog (*Phacochoerus africanus*). We calculated the CPUE by multiplying the number of independent events at each CTs by the species average mass, divided by the CTs sampling effort, and standardised per 100 camera trap days [14]. Prey mass was based on standard reference guides [43]. The CPUE index is often used in the fisheries industry to assess stock abundance, and provide information for monitoring the effects of harvesting on populations [44, 45].The concept behind CPUE is that the size of the catch from a population should increase when population density or effort increases [46]. Thus, in principle, CPUE could serve as an abundance index, and be used to detect variation in numbers as in abundance itself. The concept has been used in the studies of carnivore live trapping [47], bushmeat harvesting and poaching [48, 49], and to estimate prey biomass in camera-trapping and occupancy studies [50]. We also calculated a livestock presence covariate by summing the total independent livestock detections at each CTs. Livestock species included cattle, goats, and donkeys. We pooled these species because the objective was to assess the overall disturbance potential of livestock grazing on leopard site use, irrespective of the livestock species. We considered independent detection events for leopard, prey and livestock as those with > 5 minutes between records [14].

**Table 1:** Covariates and corresponding expected influence on the estimates of leopard site use and detection in the Ruaha landscape, southern Tanzania, during the dry seasons of 2014–2015. *Ψ*: *Estimated* probability of site use; *p*: probability of detection, given site use. CPUE: catch-per unit effort index of prey availability for each camera-trap station based on the number of independent records for the main five leopard prey species [42] photographed during the survey.

Covariates	Model type	Expected influence
Livestock presence	*Ψ*	-
Distance to Greater Ruaha River	*Ψ*	+
Distance to household	*Ψ*	+
Prey availability (CPUE)	*Ψ*	+
Trail type	*p*	+

Given that trail types have been found to influence on probability of carnivore detection in this study region [31], we evaluated the effect of trail type [animal trails (AT); no-trails (NT); human-made roads (RD)] on leopard detection probability.

Prior to model fitting, we standardized (z-score) all covariates [51], and assessed predictor collinearity using Pearson correlation and variance inflation factor tests. All the covariates used in the models were those minimally correlated (Pearson <0.7, VIF <3 [52]; S1 and S2 Tables.).

### Model analyses and averaging

We used temporally replicated surveys (i.e. weeks) to estimate the latent, unobserved probability of site use of each CT, *Z*_*i*_, where *Z*_*i*_ = 1 if site *i* is occupied and 0 otherwise. We used the replicate surveys to estimate detection probability, *p*_*i*,*j*_, where *p*_*i*,*j*_ is the probability that leopards are detected at site *i* during replicate *j*, given use of that site (i.e., *Z*_*i*_ = 1) [33, 53]. We fit the model with a random intercept at the level of each of the 11 areas sampled in the study [54, 55] to minimise potential spatial autocorrelation among model residuals (S2 Fig). Our final model to estimate leopard site use was implemented as follows:

$$\mathrm{logit}\left(\Psi_{i}\right){=\alpha }_{\mathrm{area}}{+\alpha }_{1}{*\mathrm{Livestock}\;\mathrm{presence}}_{i}{+\alpha }_{2}{*\mathrm{Distance}\;\mathrm{to}\;\mathrm{Great}\;\mathrm{Ruaha}\;\mathrm{River}}_{i}{+\alpha }_{3}{*\mathrm{Distance}\;\mathrm{household}}_{i}{+\alpha }_{4}*\mathrm{Prey}\;\mathrm{availability}\;{\left(\mathrm{CPUE}\right)}_{i}$$ (1)

where *Ψ*_i_ represents the probability of leopard site use at the *i*^th^ CT, *α*_area_ represents a random intercept indexed by sampling area with estimated hyperparameters *μ* (mean) and τ^2^ (variance), and *α*_*1*,*2…5*_ represent the influence of associated covariates at the *i*^th^ CT (Table 1).

The final detection model was implemented as follows:

$${\mathrm{logit}(p}_{i,j}{)=\beta }_{0}{+\beta }_{k}{*\mathrm{Trail}}_{i}$$ (2)

where *p*_*i*,*j*_ represents the probability of detection at the *i*^th^ CT during survey *j* given that a site is used (i.e., *Z*_*i*_ = 1), *β*_*0*_ is the intercept, and *β*_k_ represents the effect of the *k*^th^ trail type (*k* = 3) on leopard detection at each CT, with animal trail (AT) as the reference category.

We implemented and analysed the models using a Bayesian framework and Markov chain Monte Carlo (MCMC) simulations in R v.2.13.0 [56] and JAGS [57] through the package ‘R2jags’ [58]. We estimated the degree of support for the effect of each covariate on site use through the Bayesian inclusion parameter w_c_ [59], which had a Bernoulli distribution and an uninformative prior probability of 0.5. The posterior probability of w_c_ corresponds to the estimated probability of any given covariate (‘C’) to be included in the best model of a set of 2^C^ candidate models [14, 55, 60]. We calculated model-averaged estimates for the covariate coefficients over the global models from MCMC posterior histories, as described by Royle & Dorazio [60]. We used uninformative uniform priors for all covariates and implemented the models using three chains of 500 000 iterations each, discarding the first 50 000 as burn-in, and thinned the posterior chains by 10. We assessed the model convergence by ensuring R-hat values for all parameters was <1.1 [61].

## 3. Results

We recorded a total of 232 independent leopard events over 12 987 camera-trap days at 42 of the 127 CTs (33%). We recorded 197 leopard detections at 36 out of 77 CTs in the national park, 35 detections at 6 out of 16 CTs in WMA, and no detections at the 35 CTs installed in the village lands, despite the consistent sampling effort in this area (Fig 1; Table 2).

**Table 2:** Sampling effort per area in the Ruaha landscape, southern Tanzania. CT effort (days): Number of active days of survey.

Land-management	Area	CT effort (days)
	Kwihala	196	
	Lunda-Ilolo	196	
National Park	Mdonya	226	
	Msembe	7,447	
	Mwagusi	173	
	Lunda	867	
Wildlife Management Area	Pawaga	738	
	Idodi	674	
Village land	Magosi	656	
	Malinzanga	718	
	Nyamahana	1,059	

**Fig 2:**
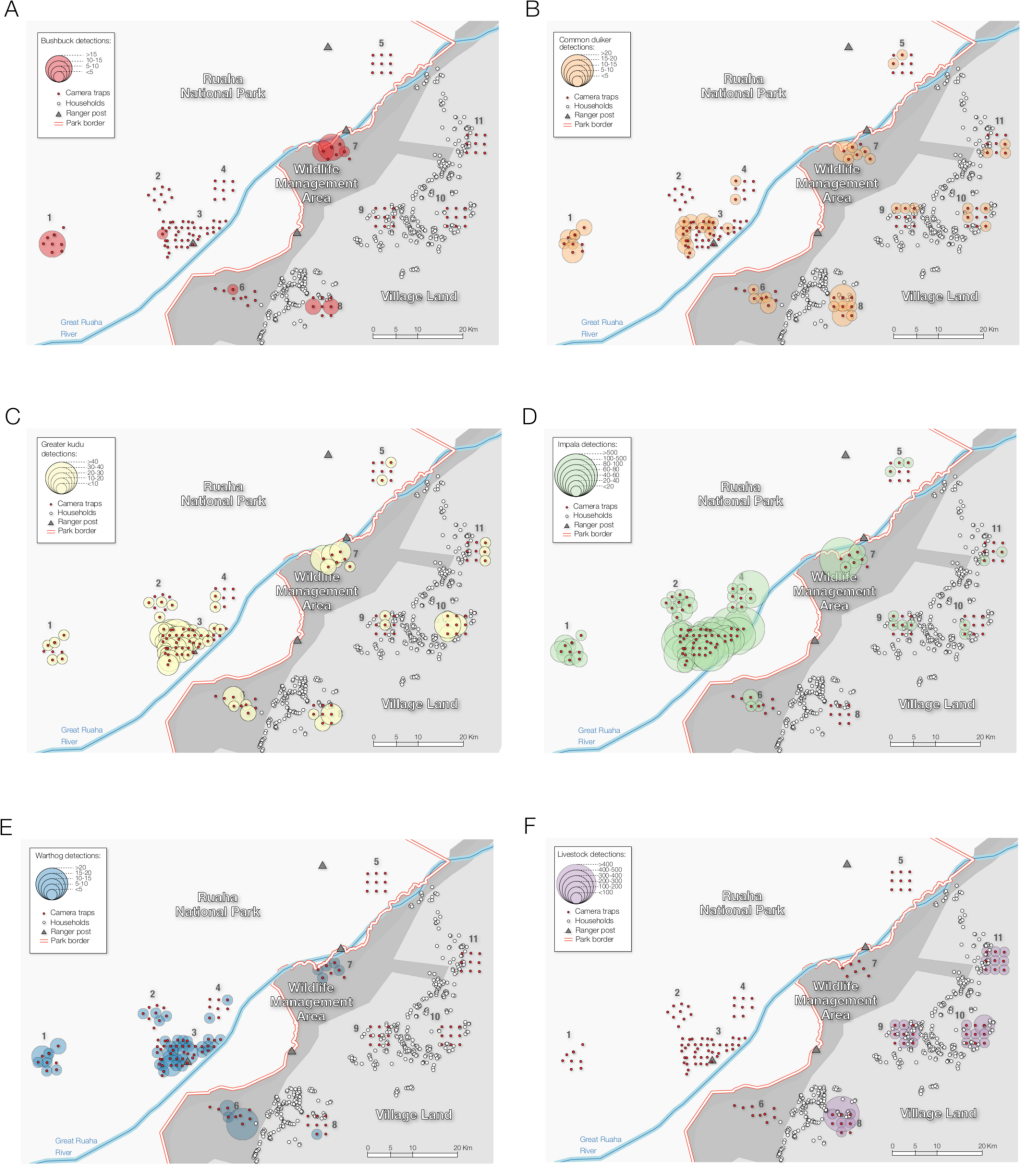
Independent detections of the main leopard prey species at each camera-trap station. A. Bushbuck (*Tragelaphus scriptus*); B. Common duiker (*Sylvicapra grimmia*); C. Greater kudu (*Tragelaphus strepsiceros*); D. Impala (*Aepyceros melampus*); E. Warthog (*Phacochoerus africanus*); F. Livestock. 1–11 represents sampling areas: 1. Mdonya; 2. Kwihala; 3. Msembe; 4. Mwagusi; 5. Lunda-Ilolo; 6. Pawaga; 7. Lunda; 8. Idodi; 9. Malinzanga; 10. Nyamahana; 11. Magosi.

**Fig 3:**
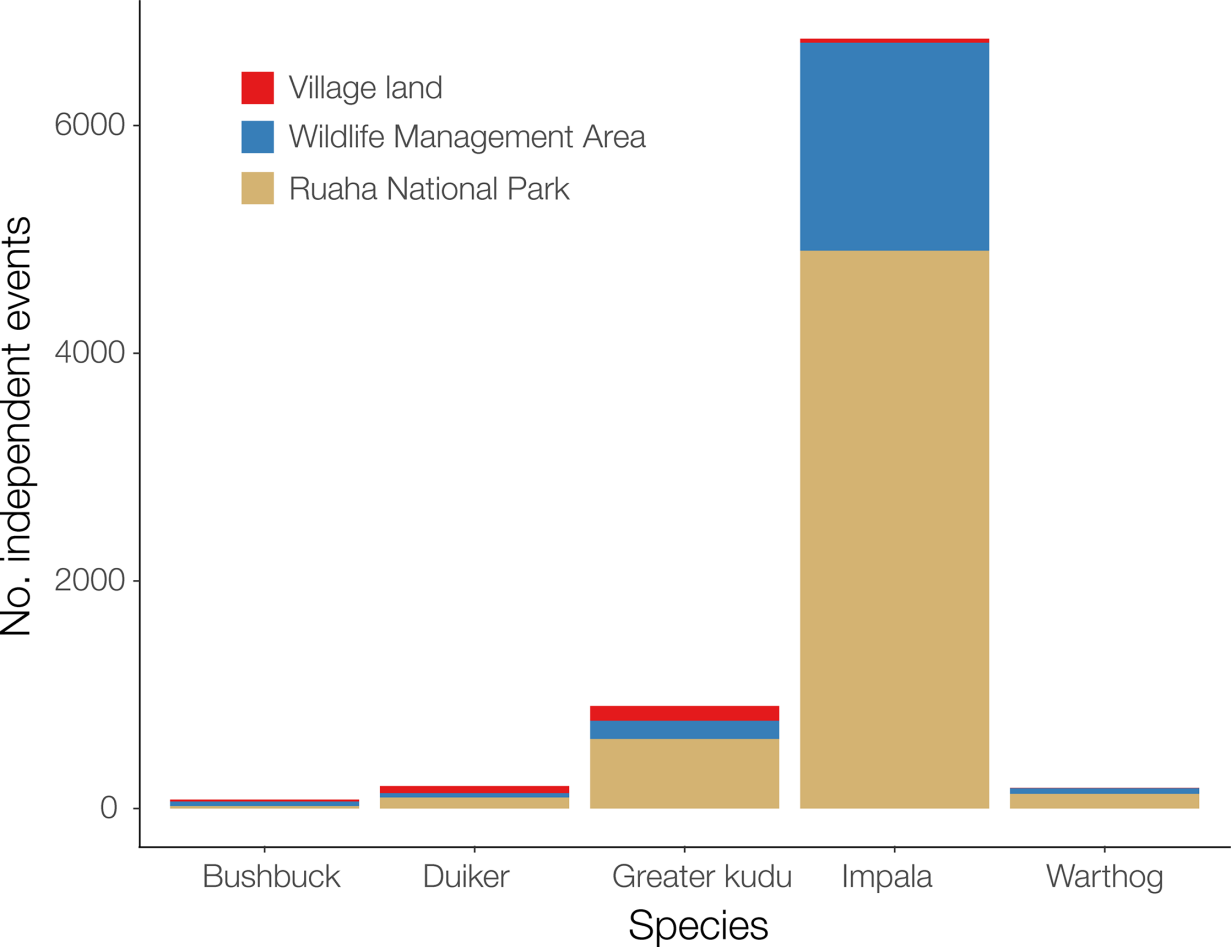
Variation in prey detection across the gradient of anthropogenic pressure in the Ruaha landscape. Independent events (> 5 min interval between detection). Bushbuck (*Tragelaphus scriptus*); Common duiker (*Sylvicapra grimmia*); Greater kudu (*Tragelaphus strepsiceros*); Impala (*Aepyceros melampus*); Warthog (*Phacochoerus africanus*).

We recorded a total of 8 120 independent detections of the primary prey of leopards (Figs 2 and 3). We observed spatial variation in the number of primary prey detections, with a total of 5 766 independent prey records in the national park, 2 116 in the WMA, and 238 in the village lands (Fig 3). We registered 2 811 independent events of livestock in 32 out of 35 village land CTs.

**Fig 4:**
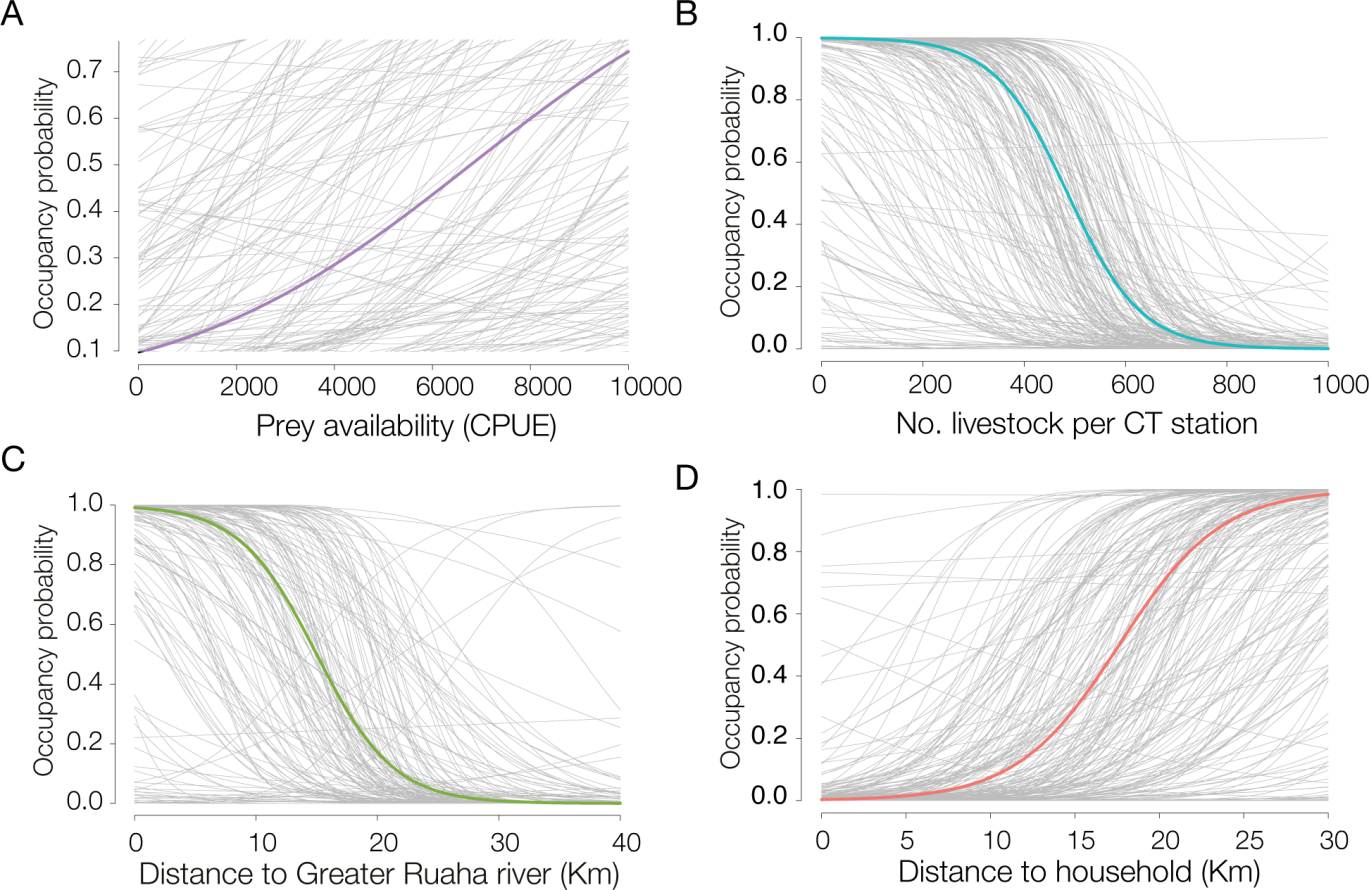
Predicted association of the covariates to the probability of site use of leopards (*Panthera pardus*). The solid line represents the posterior mean, and the light grey lines represent the estimated uncertainty based on a random posterior sample of 150–200 iterations. Occupancy probability = site use.

We found a significantly strong negative relationship between the probability of leopard site use and habitats that were closer to households. Similarly, we observed a negative, albeit highly variable and non-significant, influence of increased livestock presence on leopard site use. Additionally, we found no evidence for a relationship between prey availability, distance to the Great Ruaha River, and the probability of leopard site use (Table 3; Fig 4). The relatively high Bayesian inclusion parameter values (wc−Table 3) for both proximity to households and livestock presence, in comparison to prey availability, suggest that leopard site use was primarily influenced by lower levels of anthropogenic pressure than prey availability during the survey. Finally, we found a lack of effect of trail type on detection probability (Table 3).

**Table 3:** Posterior means, standard deviations (S.D.), 95% credible intervals (C.I.), and Bayesian inclusion parameters (w_c_) of leopard site use models fit to camera-trap data from the Ruaha landscape, southern Tanzania, during the dry seasons of 2014–2015.

Covariates	Parameter	Mean	S.D.	95% (C.I.)	w_c_
Livestock presence	α_1_	-5.5	2.97	-9.82, 0.09	0.47
Distance to Great Ruaha River	α_2_	-1.94	1.66	-5.66, 1.27	0.33
Distance to households	α_3_	2.96	1.48	0.46, 6.31	0.73
Prey availability (CPUE)	α_4_	0.62	0.58	-0.23, 2.04	0.1
Intercept	β_0_	-1.59	0.09	-1.77, -1.41	NA
Trail type N	β_k2_	-0.21	0.45	-1.05, 0.61	0.01
Trail type RD	β_k3_	-0.55	0.39	-1.4, 0.15	0.01
Estimated number of sites used	Ψ*-	51.83	4.02	45, 60	NA

* Denotes estimated number of sites used out of all surveyed sites.

## 4. Discussion

Our findings suggest that human-related activities such as increased livestock presence and proximity to human households exerted stronger influence than prey availability on leopard site use, and were the major limiting factors of leopard distribution across the gradient of human pressure, especially in the village lands outside Ruaha National Park. Leopards have been shown to adapt to heavily disturbed anthropogenic environments, occurring in areas with high human densities and of low wild prey density [19, 62, 63]. Importantly, our results suggest that such adaptations to human-pressure and threats may be context-specific [64]. It is crucial to highlight that the limited leopard site use observed outside the national park should not be interpreted as a result of the covariates considered in this study in isolation, but also as a consequence of the underlying high persecution and human induced mortality of large carnivores in the study site [28, 65]. The combination of these factors is likely limiting leopard occurrence outside the protected area in the Ruaha landscape.

### Determinants of leopard site use

The lack of leopard detections in the village lands suggests low population densities for the species in the unprotected areas surrounding Ruaha National Park. This area has undergone rapid conversion of habitats due to intense human and livestock encroachment [66, 67], intense conflict and high human-induced carnivore killing [28, 65], with all these factors likely contributing towards creating a hard edge for leopard populations in these non-protected areas. These results are similar to those presented by Henschel et al. [68] and Ramesh et al. [69], that found leopard use of habitat and abundance to be negatively influenced by areas with high human activity or increased bushmeat poaching. The negative influence of livestock presence on leopard site use could suggest a potential risk-avoidance strategy targeted at areas of intense human exposure. Large carnivores have been found to change and adjust spatiotemporal behaviour and home range in areas of intense herding activities to minimise exposure to human herders and livestock [70, 71]. Alternatively, intense livestock herding could be associated with overgrazing and potential displacement of wild prey across the village lands, although our analyses showed little support for this hypothesis. It is noteworthy that the lack of leopard detections in village lands should not be understood as the absence of the species in these areas. Leopards undoubtedly use these village lands, as indicated by reported livestock depredations, and the corresponding number of leopard killings in this area [28]. We acknowledge that the precise mechanistic connections between low-levels of leopard detections in the village lands and the variety of sources of anthropogenic pressure are elusive. In addition, our sampling strategy might have influenced our ability to capture habitat heterogeneity for particular covariates (e.g. livestock presence and distance to households), which could be limiting detection probability, and the models to precisely estimate the effect of such covariates on leopard habitat use. The limitations of camera-traps to only survey relatively small areas, associated with the likely low leopard densities and detection probability in village lands, means that broader survey across the whole landscape, and the use of complementary methods such as spoor tracks could render a more precise estimate of the variables influencing leopard site use in these areas of human occupation. Furthermore, due to lack of available data, we did not account for leopard movement pattern and home-range variation across the landscape and between seasons, which may have contributed to limit our site use estimates. These factors have been recently shown to substantially influence on species detection and site occupancy estimates from camera-trapping studies [72, 73]. Thus, further work based on camera-trapping should, whenever possible, incorporate movement data to improve site use and occupancy estimates. Despite these limitations, our results are the first to investigate the environmental determinants of leopard site use across the gradient of anthropogenic pressure in the Ruaha landscape, and provide much needed data to help furthering our understanding of the effects of human activities on limiting leopard spatial distribution across one of the most important large carnivore strongholds in East Africa.

The observed weak association between leopard site use and primary prey availability (Table 3; Fig 4) provided an interesting insight into leopard ecology in this landscape. Prey availability is a known determinant of site use, spatial distribution, and population density of leopards [15, 38, 74] and other carnivores [22, 75, 76]. In fact, recent studies have shown that areas of increased leopard population density were linked to high abundance of medium-sized wild prey [15, 69]. One explanation of the observed weak relationship is that leopards could be relying on smaller prey species than those considered in this study, especially outside Ruaha National Park, as a potential response to larger prey scarcity. Leopards are known to shift and rely on small-sized prey species (<20 kg) in areas of increased bushmeat hunting and intense competition with humans for limited food resources [42, 68, 77], similar to those of the village lands around the national park. The low prey detection across village lands, where they are exposed to intense bushmeat poaching [78], could help to corroborate such hypothesis (Figs 2 and 3). Even though we found weak association between leopard site use and prey availability, it is nonetheless important to highlight that prey depletion could still pose a serious threat to leopards locally. Prey depletion is one of the main limiting factors to leopard occurrence and population density across their extant range, and potentially more detrimental to their survival than direct human-induced killings [15, 19, 69].

### Implications for leopard conservation

Our results highlight the importance of protected areas on the conservation of wide-ranging large carnivores such as the leopard. Large protected areas such as Ruaha National Park are fundamental in protecting important habitats for leopards and other large carnivores [79, 80] against the increasing human pressure observed in village lands surrounding protected areas across Africa [8, 9, 66]. Our findings suggest that intense human activities, likely coupled with underlying high levels of human-induced carnivore mortality due to conflict [28, 36, 81], represent key-limiting factors to leopard spatial distribution in the human-dominated non-protected areas around Ruaha National Park. Similar results have been found elsewhere in Africa, where the spatial distribution and population density of leopards [15], as well as of other large carnivores such as lions [82] and other smaller carnivores [14, 83] have been limited by increased human and livestock encroachment, pastoralism, conflict and human-mediated mortality in anthropogenic landscapes surrounding protected areas. If leopards are to be successfully conserved in such areas of human occupation, it is vital to address the threats imposed by people and livestock immediately adjacent to protected areas.

In the context of this study, one much-needed strategy is the mitigation of carnivore-related conflict with people [28, 65, 81]. Increasing people’s awareness and access to effective actions to reduce the perceived hazard originating from carnivore presence could help to increase tolerance and improve attitudes towards leopards and other large carnivores locally [84]. For example, systematic widespread improvement of husbandry practices using predator-proof bomas [81, 85], and prevention of human-carnivore conflict could lead to a substantial reduction in leopard and other large carnivore mortality, and contribute to conservation of these species in the village lands [65]. Additionally, developing strategies to reduce the associated costs of large carnivores’ presence while increasing the tangible benefits of having these species in the village lands could help to promote their conservation [65, 86, 87]. For instance, the provisions of veterinary medicines, health care, and education associated with large carnivore presence as part of a community-based conservation approach in some of the villages around Ruaha National Park resulted in 80% decline of large carnivore killing, although those initiatives currently operate across less than half of the village land [65].

On a landscape level, concerted efforts to develop integrated management strategies and adaptive livestock and wildlife foraging systems could help limit the impact of livestock on rangeland habitats and wildlife [88]. Guaranteed access to optimum foraging sites by livestock, and the implementation of planned grazing strategies–which consists of establishing several grazing paddocks that enable livestock rotation based on forage growth rate—across rangelands could help minimising competition with wildlife, prey depletion, habitat degradation due to overgrazing, and ultimately promote wildlife conservation [88, 89]. However, these strategies can be difficult to implement in areas where livestock owners can be highly nomadic and transient, as is the case in the vicinity of Ruaha National Park. Finally, we emphasize that strategies aimed at conserving leopards and other large carnivores within human-dominated lands should be implemented in collaboration with local communities, given that these local communities will bear the costs of co-existing with these species, and ultimately be responsible for deciding upon their conservation [90].

## Supporting information

S1 Fig Set of covariates hypothesised to influence site use by leopards (*Panthera pardus*) across the Ruaha landscape, southern Tanzania, during our surveys in the dry seasons of 2014–2015. A. Distance to the Great Ruaha River; B. Distance to households. Primary prey availability (CPUE), livestock presence and trail type not represented here.
(TIF)

S2 Fig Spline correlograms for the leopard (*Panthera pardus*) occupancy models. Spline correlograms from a generalized linear model (A) and a generalized linear mixed model that included a random intercept at the CT level (B) showing a reduction in spatial autocorrelation. Distance between paired sample locations in kilometres (Km).
(TIF)

S1 Table Pearson’s correlation of the putative ecological variables used to model leopard (*Panthera pardus*) site in the Ruaha landscape, southern Tanzania. Dist.: distance.
(DOCX)

S2 Table Variance inflation factor (VIF < 3) of the ecological covariates used to model site use by leopards (*Panthera pardus*) in the Ruaha landscape, southern Tanzania. (DOCX)
