# Supplementary figures and images for: Spatial variation in leopard (Panthera pardus) site use across a gradient of anthropogenic pressure in Tanzania's Ruaha landscape
Source: PLoS One. 2018 Oct 10;13(10):e0204370. doi: 10.1371/journal.pone.0204370 (PMC6179245; doi:10.1371/journal.pone.0204370)

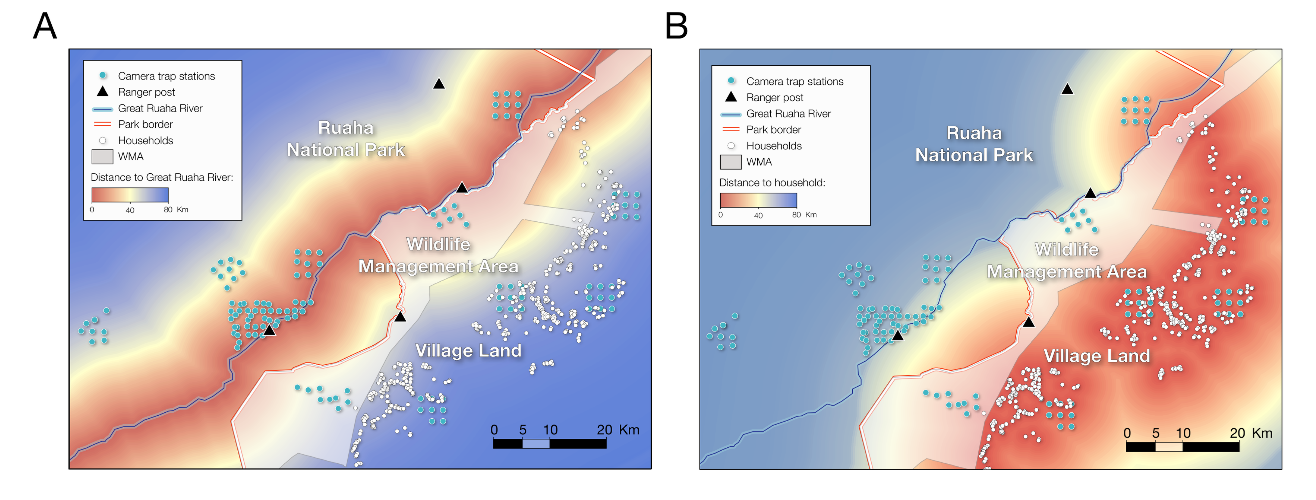

Supplement: S1 Fig — A. Distance to the Great Ruaha River; B. Distance to households. Primary prey availability (CPUE), livestock presence and trail type not represented here. (TIF) [file pone.0204370.s001.tif]

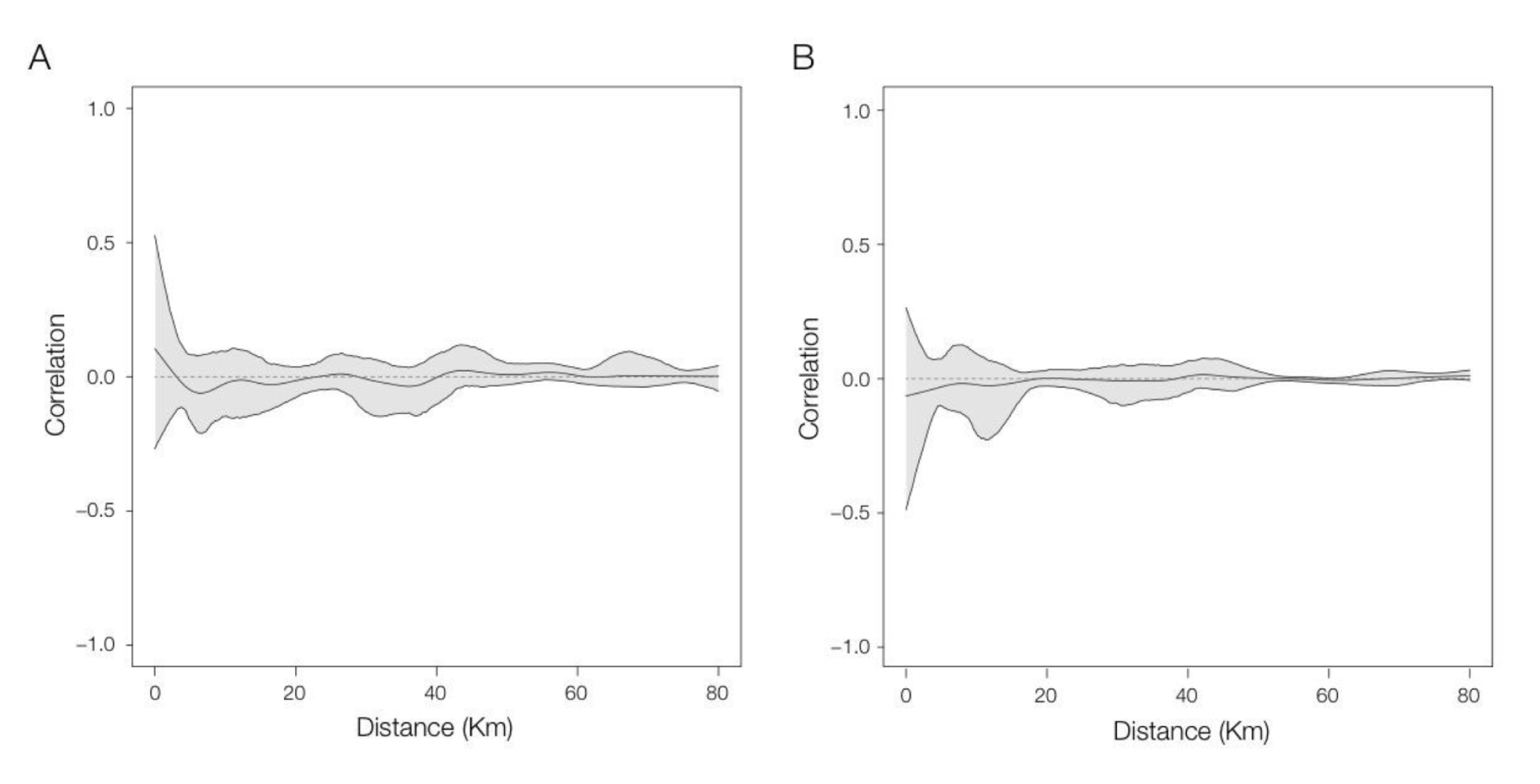

Supplement: S2 Fig — Spline correlograms from a generalized linear model (A) and a generalized linear mixed model that included a random intercept at the CT level (B) showing a reduction in spatial autocorrelation. Distance between paired sample locations in kilometres (Km). (TIF) [file pone.0204370.s002.tif]
