# Supplementary material for: Spatial variation in leopard (Panthera pardus) site use across a gradient of anthropogenic pressure in Tanzania's Ruaha landscape
Source: PLoS One. 2018 Oct 10;13(10):e0204370. doi: 10.1371/journal.pone.0204370 (PMC6179245; doi:10.1371/journal.pone.0204370)
Supplement: S1 Table — Dist.: distance. (DOCX) [file pone.0204370.s003.docx]

| **S1 Table**. Pearson’s correlation of the putative ecological variables used to model leopard (*Panthera pardus*) site in the Ruaha landscape, southern Tanzania. Dist.: distance | | | | |
| --- | --- | --- | --- | --- |
| **Ecological Covariates** | Dist. household | Dist. Greater Ruaha river | Livestock presence | Prey availability (CPUE) |
| Distance to household | 1 | - | - | - |
| Distance to Greater Ruaha river | -0.43 | 1 | - | - |
| Livestock presence | -0.18 | 0.86 | 1 | - |
| Prey availability (CPUE) | 0.38 | -0.26 | -0.09 | 1 |
