# Supplementary material for: Spatial variation in leopard (Panthera pardus) site use across a gradient of anthropogenic pressure in Tanzania's Ruaha landscape
Source: PLoS One. 2018 Oct 10;13(10):e0204370. doi: 10.1371/journal.pone.0204370 (PMC6179245; doi:10.1371/journal.pone.0204370)
Supplement: S2 Table — (DOCX) [file pone.0204370.s004.docx]

| **S2 Table.** Variance inflation factor (VIF < 3) of the ecological covariates used to model site use by leopards (*Panthera pardus*) in the Ruaha landscape, southern Tanzania. | |
| --- | --- |
| **Ecological covariates** | **VIF** |
| Distance to household | 1.36 |
| Distance to Greater Ruaha river | 1.27 |
| Livestock presence | 1.05 |
| Prey availability (CPUE) | 1.18 |
